# Surgical Morbidity and Lung Function Changes After Laser–Assisted Pulmonary Metastasectomy: A Prospective Bicentric Study

**DOI:** 10.3389/fsurg.2021.646269

**Published:** 2021-06-01

**Authors:** Mohamed Hassan, Thomas Graeter, Irene Dietrich, Lars Johann Kemna, Bernward Passlick, Severin Schmid

**Affiliations:** ^1^Department of Thoracic Surgery, Medical Center – University of Freiburg, Freiburg, Germany; ^2^Comprehensive Cancer Center Freiburg, Medical Center – University of Freiburg, Freiburg, Germany; ^3^Faculty of Medicine, University of Freiburg, Freiburg, Germany; ^4^Department of Thoracic Surgery, Klinik Loewenstein, Loewenstein, Germany; ^5^Department of Radiology, Medical Center – University of Freiburg, Freiburg, Germany

**Keywords:** morbidity, outcome, pulmonary metastasectomy, laser-assisted, lung function, laser

## Abstract

**Objective:** The surgical resection of pulmonary metastases is associated with a survival benefit in selected patients. The use of laser devices for pulmonary metastasectomy (PM) is believed to facilitate the complete resection of metastases while preserving a maximum of healthy parenchyma. This is a prospective study to evaluate surgical outcome including the changes of lung function after laser–assisted surgery (LAS).

**Methods:** A total of 77 operations in 61 patients in which PM was carried out in a curative intent were analyzed. A 1.320 nm diode-pumped Nd: YAG-Laser was used for resection of the metastases. Surgical and clinical data were collected using a standardized form and postoperative lung function changes 3 and 6 months after surgery were assessed using whole body plethysmography and diffusion capacity for carbon monoxide (DLCO). Size and distance of metastases to the pleural surface were measured radiologically.

**Results:** A median of two metastases were resected per operation (range 1–13). The median duration of postoperative air leak was 1 day (range 0–11). LAS associated postoperative minor and major complications were observed in 4 (5%) cases and 1 (1%) case, respectively; there were no mortalities. The analysis of perioperative lung function showed that mean VC 3 months after surgery was reduced by 11 %, FEV1 by 11% and median DLCO by 11% (all *p* < 0.0001). There was almost no recovery of lung function between 3 and 6 months in the whole cohort. Patients with two or less metastases showed a recovery of lung function after 3 months regarding DLCO (*p* = 0.003). Decline of DLCO in the whole cohort correlated with the number of resected metastases at 3 months (*r* = 0.45, *p* = 0.006) and at 6 months (*r* = 0.42, *p* = 0.02) as well as depth of metastases in the parenchyma at 6 months (*r* = 0.48, *p* = 0.001).

**Conclusions:** LAS is a safe and effective method for PM even for higher numbers of metastases, with short duration of postoperative air leak and little morbidity. Number and depth, but not size of metastases affect lung function changes after resection.

## Introduction

Pulmonary metastasectomy was associated with a survival benefit in selected patients in numerous retrospective trials; however, survival benefit has yet to be proven in prospective randomized studies (1–8). Different surgical techniques are used for PM including staplers, cautery devices, Ligasure/Ultracision-System as well as Nd:YAG-Lasers. Laser-assisted-resection (LAS) is believed to facilitate the complete resection of metastases is associated with long-term survival in selected patients (2, 3, 9–11). LAS devices enable the oncologic surgeon to resect lesions with high precision causing only minimal deformity to the adjacent lung tissue and thus to preserve healthy lung tissue. Moreover, central lesions or metastases, which are localized deep in the parenchyma can be evaporized as an alternative to anatomical resection avoiding segment- or even lobectomies.

Controversies about the oncologic benefit of metastasectomy over systemic treatment alone are ongoing and treatment choice, especially in presence of higher number of metastases, often depend on the treating discipline and center. Nevertheless, local ablative therapies have now been shown to prolong survival in metastatic disease in randomized-controlled trials (12, 13). PM itself is associated with little morbidity and almost no mortalities in the generally relatively healthy patient collective. Unwanted effects of PM include hospital stay, loss of lung function and prolonged or even chronic pain. In this prospective trial we analyzed outcome parameters including lung function changes after PM using exclusively a 1320 nm Nd:YAG-Laser.

### Patients and Methods

This is a prospective bicentric single arm trial. The study was approved by the Ethics Committee of the University of Freiburg Medical Centre and registered in the German Registry for Clinical Trials (DRKS00011918). All procedures were carried out in accordance with relevant guidelines and regulations. Informed consent was obtained from all patients prior to enrollment in the trial. Between October 2017 and January 2019 a total of 61 patients who underwent pulmonary metastasectomy in curative intent were included in the study. Depending on localization and size of metastasis PM was carried out either as uni- or bilateral thoracotomy or, when feasible and a complete resection of metastases could be ensured, thoracoscopy. Lung tissue was resected using a 1.320 nm diode-pumped Nd:YAG-Laser (Limax® 120, Gebrüder Martin GmbH & Co. KG, Tuttlingen/Germany). Due to the high water content of lung parenchyma, the particular wavelength is able to simultaneously cut, coagulate and seal the tissue. The focusing handpiece has to be used in non-contact mode with a focal distance of 30 mm for resection or defocused, with a distance of >30 mm, for coagulation. An integrated air-cooling was used to blow away the smoke. In thoracoscopic resection a fiber was used with special guiding instruments in non-contact mode with a short distance of a few millimeters between fiber tip and lung tissue. The smoke created during the laser resection was evacuated with the smoke suction device integrated in the laser. All resulting defects in the lung parenchyma were closed by running suture using monofilament absorbable sutures.

Perioperative parameters were collected using a standardized form: Intraoperative blood loss, duration of surgery, placed chest tubes, used energy and postoperative parameters including drainage time, total pleural-effusion, duration of air leakage, as well as postoperative complications. Complications were graded using the Clavian-Dindo-classification: grade I and II are considered as minor and grade III and IV as major complications (14). Resection status was documented, however due to the nature of evaporization and the consequent lack of a pathologic specimen could not be reliably assessed in some patients.

All preoperative computed tomographies were assessed for number, depth and size of metastasis by a board certified radiologist (ID).

Lung function was measured using whole body plethysmography and diffusion capacity for carbon monoxide (DLCO) preoperatively as well as 3 and 6 months after the last operation in case of bilateral resection. Ten patients were lost to follow-up and there were no lung function tests carried out postoperatively. These values were excluded from the analysis. At 3 months in 44 patients and at 6 months 38 patients lung function tests were carried out. Moreover two measurements were excluded from the analysis as they showed a significant improvement (>30% of DLCO and/or VC) at 3 or 6 months postoperatively which was interpreted as a false initial measurement.

### Statistics

Statistical analysis was carried out as described before (15). Data were recorded in a database designed in Microsoft Office Excel (Microsoft, Redmond, WA, USA) and GraphPad Prism 8.2.1 (GraphPad Software Inc., La Jolla, CA, USA) was used for statistical analysis. Categorical and count data are presented as frequencies and percentages. Data sets were tested for normality using the D'Agostino–Pearson omnibus normality Test. In normally distributed data sets paired *T*-Test, in non-normally distributed data sets Wilcoxon matched-pairs signed rank test was performed in case of repeated measurements. In unrelated measurements, Student's *T*-Test and Mann-Whitney-Test were applied, respectively. More than two non-parametric samples were analyzed using the Kruskal-Wallis-Test. Results were considered statistically significant if the *p*-value was <0.05. A trend was considered when the *p*-value was between 0.05 and 0.1.

## Results

A total of 77 operations were analyzed in 61 patients. Seventy-four surgeries were carried out by means of thoracotomy and 3 by video-assisted thoracoscopy (VATS). Median age at the time of surgery was 64 years (range 20–88) and 43 (70%) patients were male. A median of 2 metastases were resected per operation (range 1–13). The median duration of postoperative air leak was 1 day (range 0–11 days), and median length of hospital stay was 7 days (range, 4–24). LAS associated, postoperative minor complications were observed in 4 (5%) cases, which were all pneumonia and one major postoperative complication (bleeding) was observed; there were no mortalities. In two cases a chylothorax was reported, which were interpreted as a complication associated with lymphadenectomy but not LAS. Selective or systematic lymphadenectomy was carried out in 61 (79%) operations. Anatomical resections were carried out in 3 (4%) cases and consisted exclusively of segmentectomies. Reason for anatomical resection were either localization close to central structures or size of metastases. Tumor histology of the metastases was colorectal carcinoma in 25 (41%), renal cell carcinoma in 9 (15%), sarcoma in 5 (8%) and others (melanoma, esophagus, non-small-cell lung cancer, solitary fibrous tumor of the pleura, pancreas, parotid gland, urethral carcinoma and squamous epithelial cancer of the skin) in 22 (36%) patients. Surgical characteristics are summarized in Table 1.

**Table 1:** Surgical parameters.

Operations	77
Duration of surgery, min.	129 (55–334)
Total metastases	2 (1–13)
Air leak, days	1 (0–11)
Chest tube, days	4 (1–14)
ICU stay, days	2 (1–8)
Hospital stay, days	7 (4–24)
Minor complications	4 (5%)
Major complications	1 (1%)
Mortalities	0

*Continous data are shown as median with range, count data are presented as frequencies and percentages*.

### Lung Function Parameters After PM

**Figure 1:**
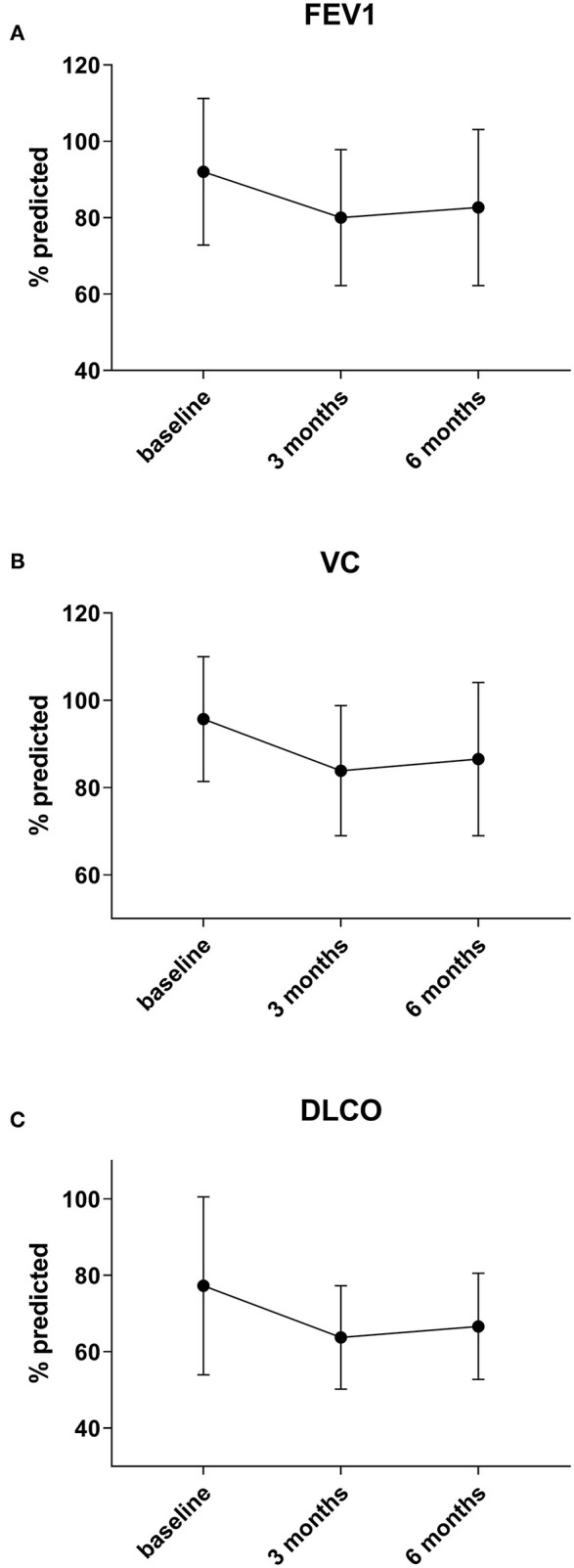
Preoperative lung function parameters show a near normal respiratory function in the cohort. Three months after surgery a decline of mean forced expiratory volume in 1 second (FEV1) by 11% **(A)**, vital capacity (VC) by 11% **(B)**, and median DLCO by 11% **(C)** (all *p* < 0.0001) is observed. There is no significant change of lung function after three months post-surgery.

Preoperative lung function parameters show a near normal respiratory function in the cohort with a mean FEV1 of 92% (± 19), VC of 96% (± 14), RV of 110% (± 29) and DLCO of 77% (± 23). Lung function analysis 3 months after surgery show a decline of mean vital capacity (VC) by 11% (*n* = 43), forced expiratory volume in 1 second (FEV1) by 11% (*n* = 44) and median DLCO by 11% (*n* = 34) (all *p* < 0.0001). There was only little recovery of lung function parameters at 6 months: VC increased by 0% (*p* = 1) (*n* = 34), DLCO by 1.5% (*p* = 0.3) (*n* = 28) and FEV1 by 0% (*p* = 1) (*n* = 35) in the whole cohort (Figure 1).

**Figure 2:**
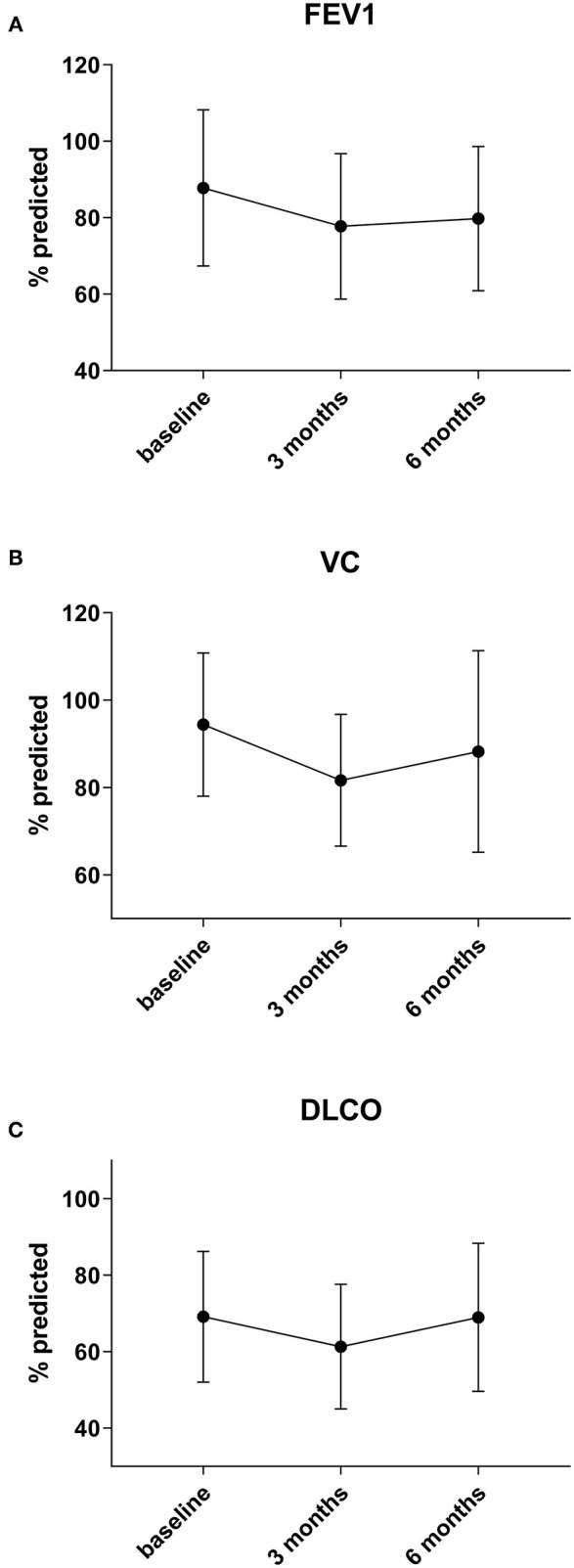
In the subgroup of patients in whom only 1 or 2 metastases were resected a significant recovery of DLCO **(C)** between 3 and 6 months by 4 % can be observed (*p* = 0.003). VC **(B)** also improves by a median of 3%, but not statistically significant, while FEV1 **(A)** shows no relevant recovery (+1, 4%) in this group (*p* = 0.26 and *p* = 0.46).

Interestingly, when analyzing the subgroup of patients with two or less metastases there is a significant recovery of DLCO from 3 to 6 months after surgery by a median of 4%, going back to baseline (*p* = 0.003) (*n* = 13). VC also improves after 3 months by a median of 3% (*n* = 16), but not statistically significant, while FEV1 shows no relevant recovery (+1, 4%) (*n* = 17) in this group (*p* = 0.26 and *p* = 0.46) (Figure 2).

### Number of Resected Metastases Affect Lung Function Changes, Particularly DLCO

**Figure 3:**
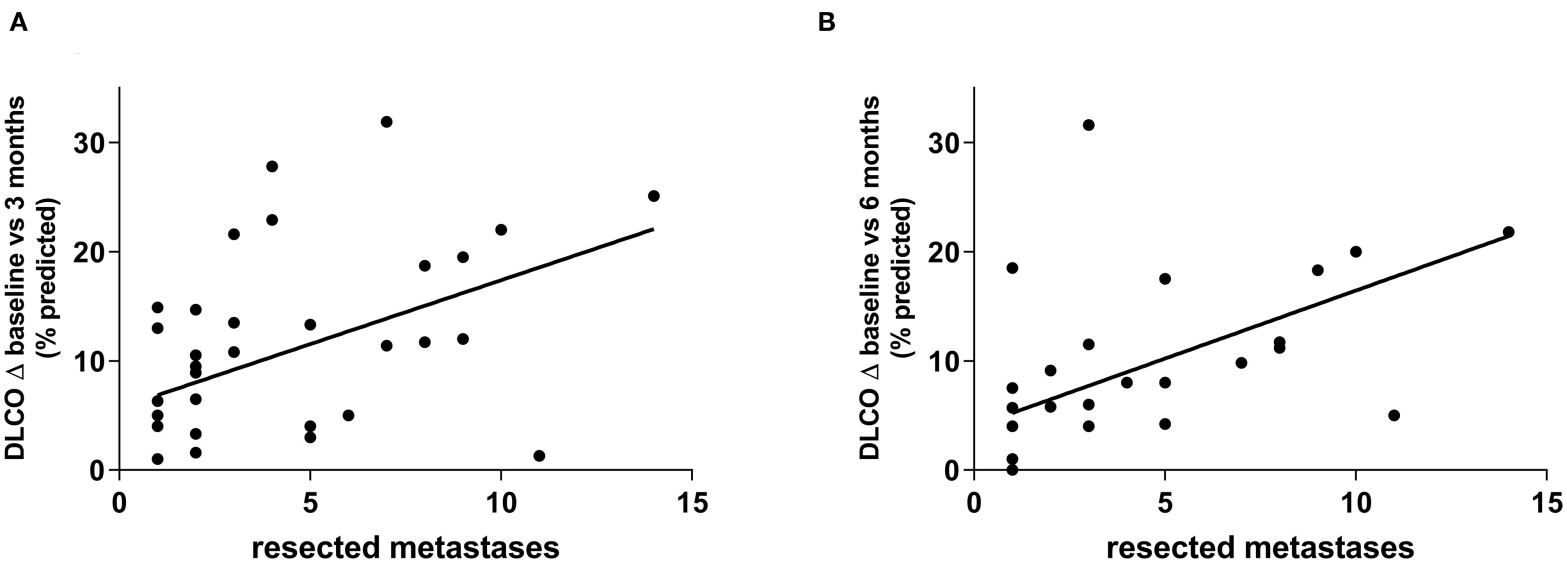
DLCO decline showed the greatest correlation with the number of resected metastases. This was observed after 3 months (*r* = 0.45, *p* = 0.006) **(A)** as well as after 6 months (*r* = 0.42, *p* = 0.02) **(B)**.

To determine the surgical parameters which influence lung function changes the most, metastases were counted and measured radiologically and correlated with the changes of lung function parameters. After 3 months, decline of DLCO correlated well-with the number of resected metastases (*r* = 0.45, *p* = 0.006) (Figure 3). Changes of FEV1 as well as RV also showed some correlation with number of metastases (*r* = 0.28, *p* = 0.07 and *r* = 0.27, *p* = 0.08) but not VC (*r* = 0.07, *p* = 0.64). Consequently, after 6 months DLCO also showed the greatest correlation with the number of resected metastases (*r* = 0.42, *p* = 0.02) (Figure 3). Changes in RV again showed some correlation but the decline of FEV1 and VC did not (*r* = 0.29, *p* = 0.07; *r* = 0.12, *p* = 0.47 and *r* = 0.11, *p* = 0.49).

**Figure 4:**
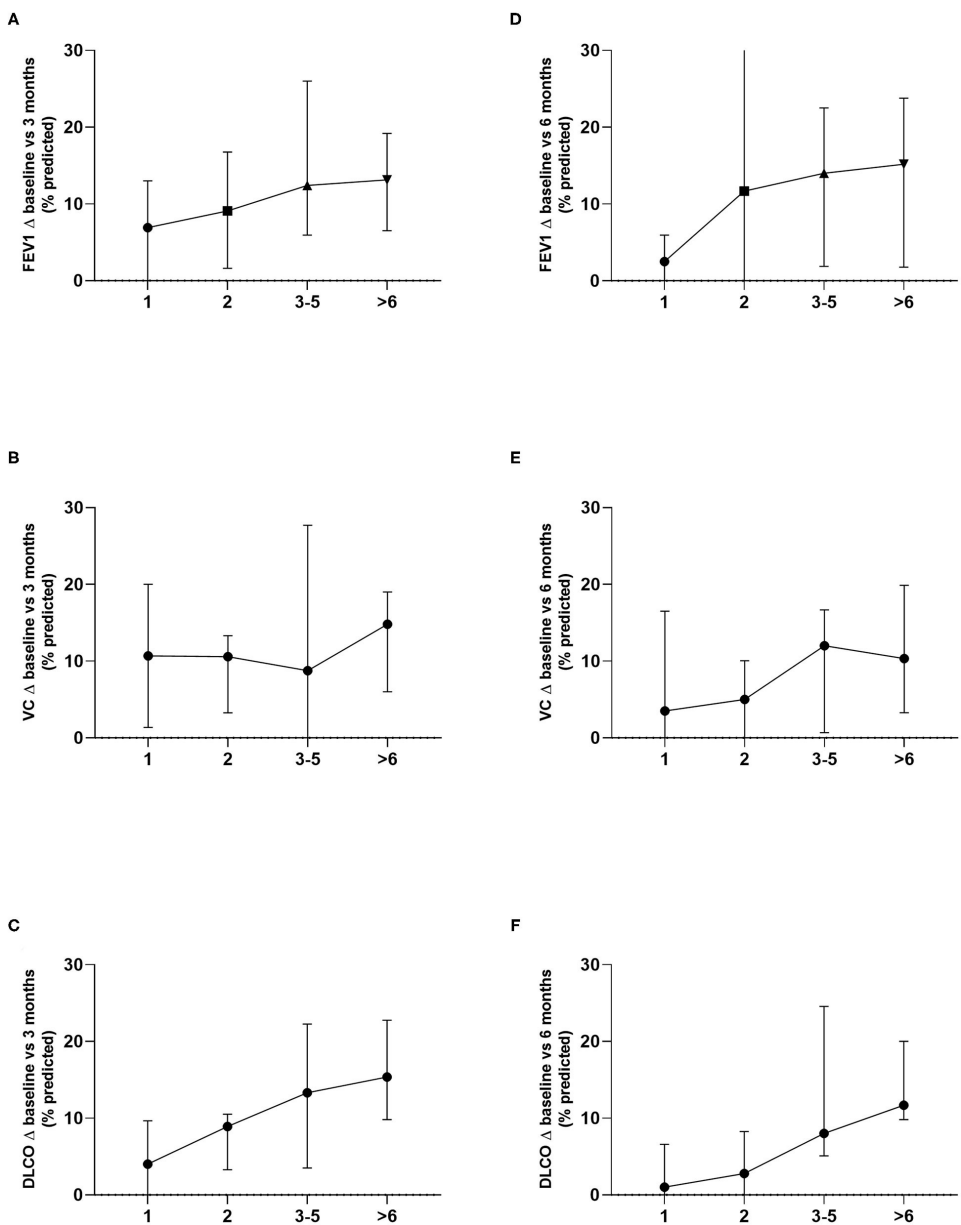
Lung function changes in the different groups according to the number of resected metastases were analyzed. Differences in decline of DLCO were the greatest and showed a statistical trend at 3 months (*p* = 0.06) and statistically significant differences at 6 months (*p* = 0.01) **(C, F)**. FEV1 **(A, D)** as well as VC **(B, E)** showed no statistically significant differences.

Moreover, we grouped patients according to the number of resected metastases in groups of 1, 2, 3-5 and 6 or more resected metastases. The greatest differences in the groups were seen for changes of DLCO with a statistical trend at 3 months (*p* = 0.06) and statistically significant differences at 6 months (*p* = 0.01). While DLCO and FEV1 at 3 months showed a consistent decline in the different groups, VC only seemed to be relevantly affected if 6 or metastases were resected. A similar observation can be made at 6 months, only here a major decline in VC is already seen at 3–5 metastases (Figure 4). Nevertheless, patient groups in these analyses are relatively small and results should be interpreted with caution.

### Depth Within the Parenchyma but Not Size of Metastases Correlate With Lung Function Changes

To determine the effect of the size and depth of metastases respective parameters were correlated with the lung function changes. Notably, the sum of the depth of the resected metastases showed a significant correlation with decline of DLCO at 6 months (*r* = 0.48, *p* = 0.001) but only a trend at 3 months (*r* = 0.28, *p* = 0.11). None of the other lung function parameters showed any correlation with the measured depth of the metastases. Moreover, the size of the resected metastases did not have an impact on the changes of lung function parameters.

### Further Parameters Affecting Lung Function Changes

Analysis on further factors affecting lung function parameters such as type of surgery, uni- or bilateral resections, previous ipsilateral surgery and presence of COPD were not carried out, as groups were too small for meaningful statistical analysis and important parameters affecting lung function changes, such as the number of metastases, differed significantly in the groups.

## Discussion

Recent studies have compared LAS to conventional PM regarding oncologic outcome and survival. Main findings of these retrospective trials were the possibility of resection of many metastases using a laser-device with good long-term results (3, 9, 11). LAS is believed to help in preserving lung parenchyma, particularly in case of resection of many metastases, by reducing the damage and deformation to the adjacent lung tissue by precise application of energy.

We evaluated lung function changes after exclusively using a laser-device for resection of metastases 3 and 6 months after surgery. Previous trials analyzing PM have shown a decline of lung function parameters immediately after surgery with a significant recovery after 3 months, no relevant changes were seen between 3 and 6 months (16–19). This is consistent with our findings regarding the whole cohort and hence does not seem to be affected by the use of the laser in comparison to other devices. Interestingly, in our trial the subgroup with two or less resected metastases shows a major recovery beyond 3 months after surgery, particularly regarding DLCO. Potentially, if only few metastases are resected regeneration of lung parenchyma is quicker and thus already seen after 6 months. However, whether there is any further recovery of lung function after 6 months is currently unknown and to our knowledge, there is no data on long-term outcome of lung function after PM.

We identify the number of metastases as the best parameter to predict the decline in lung function after PM, which goes along with previous findings (16–19). Moreover, the radiologically measured depth of the metastases correlate with lung function deterioration, particularly DLCO. Petrella et al. addressed a similar aspect in their study, where the actual extend of resection, which was defined as the total amount of resected tissue measured in the pathologic specimen, correlated with lung function changes (16). As the amount of resected tissue is majorly affected by the depth of the metastases, these findings are probably linked and the radiologic measurement can act as a surrogate for the expected loss of lung tissue. Interestingly, in our trial DLCO is the lung function parameter which shows the greatest correlation with these parameters. FEV1 as well as VC are also affected but not in a linear fashion. One can speculate that the resection using LAS in comparison to other devices rather affects the diffusion capacity than dynamic parameters; however, there are many further aspects, which influence lung function changes after pulmonary surgery, thus exact mechanisms remain unclear to this point.

Other aspects affecting lung function changes after surgery which have been identified include perioperative chemotherapy, bilateral surgery and the length of the interval between surgeries (16, 18). Unfortunately, type of surgery was not addressed in these trials as performance of a particularly bilateral thoracotomy in opposition to a minimally invasive approach will influence lung function changes. In the study at hand, this issue could also not be addressed satisfactorily, as there were only three patients in which a minimally invasive approach was performed. Previous studies, which focused on surgery requiring thoracotomy but did not involve lung resection have shown a relevant lung function decline (20–22).

In this study, we find no mortality and very little morbidity, as well as a short air leak duration, which allowed relatively early removal of the chest tube catheter. Morbidity consisted mostly of pneumonia and in one case of postoperative bleeding. Pulmonary infiltrates are regularly seen after LAS, as the adjacent tissue will react to the use of the laser or cautery devices with inflammation, therefore true pneumonia and inflammatory effects due to the laser are often difficult to differentiate. The discrepancy between the low morbidty, short air leak duration and comparatively long hospital stay is due to the German reimbursement system, which is based on diagnose-related-groups (DRG). Herein a lower length of stay threshold is determined based on different factors like performed surgery and comorbidities. Payment is reduced for every day a patient gets discharged before this predefined threshold, thus patients often stay in the hospital longer than it would be required for chest tube or pain management.

In most previous trials PM is also associated with little or no mortality, reasonable morbidity and, in selected cases, long-term survival (1–3, 8). These findings contradict the notion of surgery being essentially more harmful when compared to systemic treatment and radiotherapy. In the prospective randomized SABR-Comet trial mortality was 4.5% after stereotactic body radiation therapy which is considerably higher than in most surgical surgical series (13). As more and more trials seem to confirm the benefit of local measures in addition to systemic treatment in metastatic disease the best treatment modality has yet to be determined (12, 13, 23). Besides safety, the impact on continuity of the systemic treatment, long term quality of life and completeness of removal of the metastases have to be taken into consideration in these often complex and long treatment trajectories. As application of local ablative will most likely increase based on the recent findings, this should be assessed in a randomized trial comparing radiotherapy and surgical resection in the near future.

In conclusion, LAS is an extremely safe and effective method for PM even in high numbers of metastases, with short duration of postoperative air leak and little morbidity. LAS-PM of only few metastases results in minor lung function changes, which are largely recovered after 6 months. Preoperative assessment for sufficient cardiorespiratory reserve for PM should include the number and depth of the metastases and for LAS DLCO seems to be the lung function parameter, which shows the best correlation with surgical parameters.

## Data Availability Statement

The raw data supporting the conclusions of this article will be made available by the authors, without undue reservation.

## Ethics Statement

The studies involving human participants were reviewed and approved by University of Freiburg Ethics Commission Hugstetter Str. 55 79106 Freiburg ekfr@uniklinik-freiburg.de. The patients/participants provided their written informed consent to participate in this study.

## Author Contributions

BP and SS: conception and design. MH, TG, ID, BP, and SS: development of methodology. MH, ID, LK, and SS: acquisition of data. MH, TG, BP, and SS: analysis and interpretation of data, writing, review, and/or revision of the manuscript. TG, LK, BP, and SS: study supervision. All authors contributed to the article and approved the submitted version.

## Conflict of Interest

TG and BP have consulting contracts with KLS Martin. The remaining authors declare that the research was conducted in the absence of any commercial or financial relationships that could be construed as a potential conflict of interest.

## Supplementary Material

Supplementary Figure 1 Standardized measurement of the pulmonary metastases was performed by a board certified radiologist (ID). The size of the metastases was determined by multiplication of the long and short axis in millimeter, which were measured perpendicularly in the axial scans. The depth of the metastases was defined as the shortest distance from the pleural space to the surface of the nodule.
