# Supplementary figures and images for: Surgical Morbidity and Lung Function Changes After Laser–Assisted Pulmonary Metastasectomy: A Prospective Bicentric Study
Source: Front Surg. 2021 Jun 1;8:646269. doi: 10.3389/fsurg.2021.646269 (PMC8203914; doi:10.3389/fsurg.2021.646269)

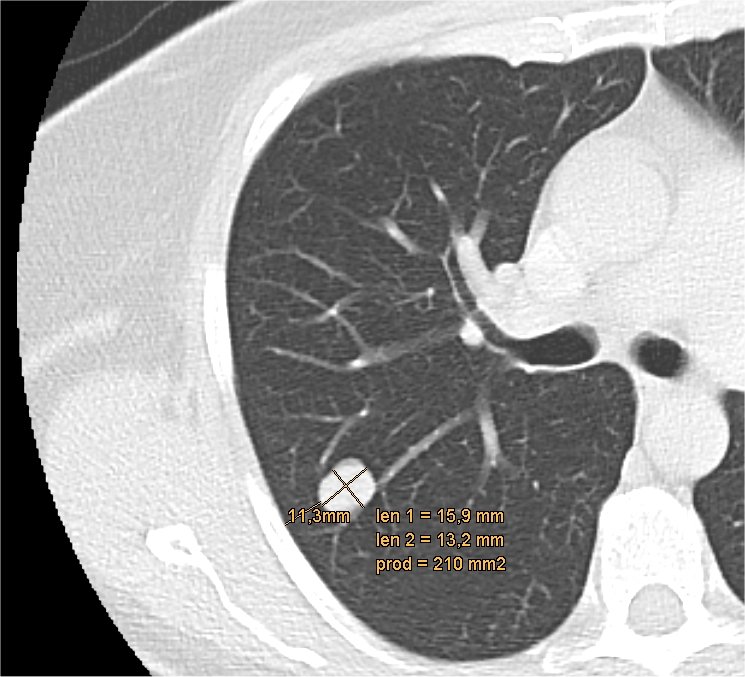

Supplement: Supplementary Figure 1 — Standardized measurement of the pulmonary metastases was performed by a board certified radiologist (ID). The size of the metastases was determined by multiplication of the long and short axis in millimeter, which were measured perpendicularly in the axial scans. The depth of the metastases was defined as the shortest distance from the pleural space to the surface of the nodule. [file Image_1.JPEG]
